# Deep Multimodal Phenotyping and Sensor Fusion for Preharvest Cotton Quality Assessment and Agricultural Economic Decision Support

**DOI:** 10.3390/s26144493

**Published:** 2026-07-15

**Authors:** Jingwen Luo, Xintong Wang, Shiguo Zhang, Jiahe Zhang, Ruobing Feng, Xiuting Shu, Shuo Yan

**Affiliations:** 1 China Agricultural University, Beijing 100083, China; 2 Peking University, Beijing 100871, China

**Keywords:** cotton boll maturity, postharvest fiber quality prediction, smart agriculture, agricultural Internet of Things, agricultural economic decision support

## Abstract

Cotton fiber quality is shaped during boll development, boll opening, fluffing, and harvesting, but current assessment still relies largely on manual field inspection and postharvest laboratory testing. This limits timely harvest scheduling and plot-level quality management. To address this problem, we propose a self-supervised multimodal sensing framework for linking preharvest cotton boll status, environmental conditions, and postharvest fiber quality. First, the Cotton Boll Visual Phenotype Self-Supervised Encoding Module learns maturity-related visual representations by reconstructing masked image patches, so that boll cracking, lint exposure, and surface texture can be captured from unlabeled field images. Second, the Agricultural Sensor Temporal Masked Modeling Module reconstructs masked sensor observations to model temporal patterns in temperature, humidity, light, soil moisture, rainfall, and other environmental variables. Third, the Vision–Environment Cross-Modal Contrastive Fusion Module aligns image features with environmental features and produces a joint representation for downstream prediction. Field experiments were conducted using cotton boll images from different maturity and abnormal states, environmental sensor records, management information, and postharvest fiber quality measurements. The framework was evaluated for maturity classification, harvest-window recognition, and fiber quality prediction. The results showed that the proposed method performed consistently better than representative machine learning, single-modal deep learning, and multimodal fusion baselines, while few-shot and ablation experiments supported the value of self-supervised pretraining and multimodal fusion. These findings indicate that the proposed approach can provide useful information for preharvest cotton maturity assessment and harvest-quality management.

## 1. Introduction

Cotton is a major natural fiber crop worldwide and remains an essential raw material for the textile industry. As modern agriculture shifts from yield-centered production toward quality- and efficiency-oriented management, fiber quality has become increasingly important for determining cotton’s economic value, textile-processing performance, and industrial supply-chain stability [1]. Producing high-quality cotton without sacrificing yield has therefore become a central concern in precision cotton field management [2]. Unlike many postharvest quality evaluation tasks, cotton fiber quality is not generated only after harvest. It is gradually formed and accumulated throughout boll development, boll opening, maturation, and harvesting [3]. During boll development, environmental conditions such as temperature, light, water availability, irrigation, and nutrient supply continuously influence fiber elongation, cellulose deposition, and maturation [4]. At the same time, visible phenotypic traits, including boll cracking degree, boll-opening status, lint color variation, and surface morphology, also change along with the maturation process [5]. These external traits provide observable evidence of the internal developmental state of cotton fiber. Accurate preharvest perception of cotton boll maturity is therefore valuable not only for identifying an appropriate harvesting period but also for supporting the prediction of postharvest fiber quality [6]. Harvest timing has a direct influence on cotton quality. Premature harvesting may lead to insufficient fiber maturity, shorter fiber length, and lower fiber strength, whereas delayed harvesting may increase the risks of wind and rainfall erosion, impurity contamination, fiber aging, and mechanical harvesting loss [7]. Thus, preharvest assessment of boll maturity and prediction of postharvest fiber quality can help optimize harvesting decisions, improve field management, and enhance the benefits of cotton production [8].

Traditional cotton quality assessment still depends mainly on postharvest laboratory testing and manual field investigation. Laboratory testing can accurately measure fiber length, fiber strength, micronaire value, maturity, trash content, and other quality indicators. However, these results are usually obtained after a clear time lag, which limits their usefulness for real-time harvesting decisions and field management [9]. Manual maturity assessment, by contrast, relies largely on empirical observation of boll cracking status and lint color. Such assessment is easily affected by observer experience, illumination, cultivar differences, plant occlusion, and field heterogeneity, which results in considerable subjectivity and instability [10]. With the development of intelligent agriculture, computer vision and agricultural sensing technologies have offered new possibilities for cotton boll maturity assessment and fiber quality prediction [11]. In recent years, deep learning has been widely used in cotton boll detection [12], growth-stage recognition [13], yield estimation, disease diagnosis [14], and automatic harvesting assistance [15]. Convolutional neural networks, object detection models, and attention-based methods are able to extract useful visual information from complex field scenes, thereby supporting intelligent cotton field management [16].

Nevertheless, existing studies still face three main limitations. First, most research has emphasized object detection, flowering recognition, or yield estimation, while the relationship between preharvest boll status and postharvest fiber quality has been insufficiently modeled. Maturity recognition and quality assessment are often handled as two separate tasks. Second, current deep learning models generally require large-scale labeled data, whereas maturity grading of cotton bolls and fiber quality testing depend on expert field investigation and laboratory analysis. The cost of annotation therefore limits the scale of high-quality datasets. Third, cotton boll maturation and fiber quality are not determined by visual phenotypes alone. They are also affected by environmental factors such as temperature, humidity, light, soil moisture, and irrigation conditions. For this reason, the mechanism of quality formation is difficult to capture using image data alone. Meanwhile, self-supervised learning has made substantial progress in computer vision, multimodal learning, and temporal modeling [17]. By learning from the intrinsic structure of data, self-supervised learning can obtain transferable representations from large amounts of unlabeled data and substantially reduce the dependence on manual annotation [18]. This feature is particularly suitable for agricultural scenarios, where unlabeled images and sensor data are relatively easy to collect but high-quality labeled samples remain limited. In addition, agricultural Internet of Things systems continuously produce large volumes of environmental monitoring data, which provide a strong data basis for self-supervised multimodal representation learning [19].

In view of these issues, a self-supervised multimodal deep sensing framework is proposed for preharvest cotton boll maturity assessment and postharvest fiber quality prediction. Large-scale unlabeled cotton boll images and agricultural sensor time series are used for self-supervised pretraining. A unified phenotype–environment joint representation is learned through visual masked modeling, temporal masked reconstruction, and cross-modal contrastive learning. Based on this representation, downstream tasks such as cotton boll maturity grade recognition, optimal harvesting window determination, and fiber quality indicator prediction can be performed with only a small number of labeled samples.

The main contributions of this study are summarized as follows:

1. A preharvest–postharvest cotton dataset is constructed by linking cotton boll images, environmental sensor records, agricultural management information, maturity labels, harvest-window labels, and postharvest fiber quality indicators at the plot or harvest-batch level.
2. A self-supervised pretraining strategy is developed for unlabeled field images and environmental sensor sequences. Masked visual reconstruction and masked sensor reconstruction are used to learn modality-specific representations, while cross-modal contrastive learning is used to align boll visual traits with environmental conditions.
3. A multimodal prediction framework is designed for three connected tasks: maturity classification, harvest-window recognition, and fiber quality regression. The framework integrates the Cotton Boll Visual Phenotype Self-Supervised Encoding Module, the Agricultural Sensor Temporal Masked Modeling Module, and the Vision–Environment Cross-Modal Contrastive Fusion Module to produce task-oriented visual–environmental representations.

The experimental section evaluates the proposed framework through maturity classification, harvest-window recognition, fiber quality regression, few-shot learning, cross-field generalization, and ablation experiments. The practical discussion further analyzes how preharvest sensing results may support harvest scheduling, quality-risk warning, graded storage, and batch-wise cotton quality management.

## 2. Related Work

### 2.1. Cotton Boll Maturity Assessment and Fiber Quality Evaluation

Cotton fiber quality plays a central role in textile processing performance and market value. Conventional cotton quality evaluation is still mainly conducted after harvest through laboratory tests, where indicators such as fiber length, fiber strength, micronaire value, maturity index, and trash content are measured. These measurements are reliable for quality evaluation, but they are obtained with an inevitable time lag, which makes them difficult to use in real-time harvesting decisions and preharvest field management [20]. For this reason, intelligent sensing technologies have been increasingly investigated for cotton boll maturity assessment and harvesting suitability evaluation [21]. Along with the progress of computer vision and deep learning, convolutional neural networks, object detection models, and Transformer architectures have been widely used in cotton production monitoring. Promising results have been reported in cotton boll detection, flowering recognition, boll-opening identification, yield estimation, and automatic harvesting assistance. For instance, Wang et al. developed a multi-task learning (MTL) framework based on the collaborative perception of remote sensing and meteorological information. By integrating climate data with Sentinel-2 remote sensing data, dynamic cotton yield prediction was achieved during the growing season, and the best performance was obtained 60 days before harvest [22]. Askr et al. introduced a cotton disease recognition framework combining explainable artificial intelligence (XAI) with Copula Entropy-based feature association analysis. ResNet50, Copula Entropy-GWO feature selection, and a random forest classifier were integrated in this framework. A disease classification accuracy of 99% was achieved, while both feature selection efficiency and model interpretability were substantially improved [23]. These studies show that cotton phenotypic information can be automatically extracted from complex field environments, which greatly improves the efficiency of cotton field monitoring [24]. In addition to object detection, several studies have used visual traits, including boll cracking degree, boll-opening status, color variation, and canopy phenotype, to assess cotton boll maturity [25]. Such visual indicators provide useful evidence for cotton boll developmental status and harvesting suitability [26]. However, most existing studies are still confined to maturity classification or harvesting status recognition, while the relationship between preharvest cotton boll status and postharvest fiber quality has received insufficient attention. Fiber quality is formed gradually during cotton boll development, whereas maturity represents only the external manifestation of this quality formation process. Therefore, when maturity assessment and fiber quality evaluation are treated as two isolated tasks, the value of existing methods for quality-oriented harvesting management is limited. In general, previous studies have confirmed the effectiveness of visual perception methods for cotton boll maturity assessment, but a direct connection between preharvest phenotypic features and postharvest fiber quality has not yet been established. A modeling framework centered on the quality formation process is therefore needed to extend maturity assessment toward fiber quality prediction.

### 2.2. Multimodal Agricultural Sensing for Crop Quality Formation Modeling

Crop quality formation is a complex biological process shaped jointly by plant phenotypes and environmental conditions [27]. Agronomic studies have shown that temperature, light, air humidity, soil moisture, irrigation management, and nutrient supply can all affect crop development and final quality [28]. In cotton production, these environmental factors influence not only boll maturation but also fiber elongation, cellulose deposition, and the formation of fiber strength [29]. Understanding cotton quality formation therefore requires the joint consideration of phenotypic information and environmental information [30].

The development of agricultural Internet of Things technologies has made multimodal sensing an important direction in precision agriculture [31]. In recent years, visual images have increasingly been combined with meteorological data, soil information, environmental sensor records, and agricultural management data to improve the performance of agricultural monitoring systems [32]. Yang et al. proposed a chlorophyll dynamic sensing framework using unmanned aerial vehicle high-throughput phenomics and multisource remote sensing fusion. RGB and multispectral images were integrated, and a random forest model was used to predict chlorophyll content and cotton yield [33]. Liu et al. proposed a machine learning diagnostic framework based on multimodal unmanned aerial vehicle sensing and leaf area index (LAI) inversion theory. By fusing multispectral, thermal infrared, and LAI information, maize water stress was accurately identified at the field scale, and cross-scenario generalization ability was significantly improved [34]. Compared with single-modal models, multimodal models can make use of complementary information from different data sources and usually achieve stronger predictive performance [35,36].

Even so, most multimodal agricultural sensing studies have focused on production management tasks, including yield estimation, stress detection, and growth status monitoring [37]. The modeling of quality formation processes has received less attention, especially with regard to the association mechanisms among environmental dynamics, phenotypic changes, and final quality. In addition, many existing methods rely on simple feature concatenation or late fusion, rather than explicitly learning the interactions between plant phenotypes and environmental driving factors. This makes it difficult to fully represent the biological mechanisms involved in quality formation. Thus, although multimodal sensing has shown strong potential in agricultural applications, the role of phenotype–environment interactions in quality prediction remains underexplored. A new representation learning framework is needed to jointly model visual phenotypes, environmental dynamics, and quality formation mechanisms.

### 2.3. Self-Supervised Multimodal Representation Learning in Agriculture

Deep learning models have delivered strong performance in agricultural sensing tasks, but their success often depends on large-scale labeled datasets [38]. In agricultural applications, high-quality labels usually require expert knowledge, field investigation, or laboratory testing, which makes data acquisition costly. For crop quality evaluation, maturity annotation and quality indicator measurement are even more complicated and time-consuming, and high-quality labeled data are therefore extremely limited. Label scarcity has consequently become a major bottleneck for agricultural deep learning [39]. In recent years, self-supervised learning has emerged as an important paradigm for learning general representations from unlabeled data [40]. In computer vision, masked image modeling and contrastive learning have shown strong representation learning ability. In temporal analysis, self-supervised methods capture temporal dependencies through sequence reconstruction and prediction tasks [41]. Multimodal self-supervised learning further aligns representations across different data sources through cross-modal contrastive learning and masked multimodal modeling, producing joint feature representations with higher quality [42].

These advances have encouraged the use of self-supervised learning in agricultural scenarios, including crop classification, disease recognition, plant phenotyping, remote sensing interpretation, and yield prediction. Zhang et al. proposed an NJ-Unet leaf segmentation framework based on interpretable deep learning and canopy porosity modeling. By integrating an SCSA attention module and an SMU activation function, high-precision estimation of tomato leaf area index and fruit yield prediction in greenhouses was achieved [43]. Choi et al. proposed a Channel Randomisation (CH-Rand) data augmentation framework based on self-supervised representation learning and color anomaly perception. A color-perturbation pretraining task was constructed to learn crop visual representations, and anomaly detection performance for specialty fruits was substantially improved. On the strawberry dataset, the AUC-PR increased by more than 43% compared with existing methods [44]. Yi et al. proposed a grape harvesting viewpoint planning framework for occluded environments based on self-supervised deep reinforcement learning. A self-supervised convolutional network and a policy network were jointly optimized to localize grape peduncles efficiently, thereby improving harvesting success rate and training efficiency [45]. Existing studies show that self-supervised pretraining can markedly improve downstream task performance under few-shot conditions. However, current agricultural self-supervised learning studies are still mainly focused on classification, detection, and segmentation, while crop quality formation modeling has received limited attention.

## 3. Materials and Method

### 3.1. Data Collection

**Figure 1:**
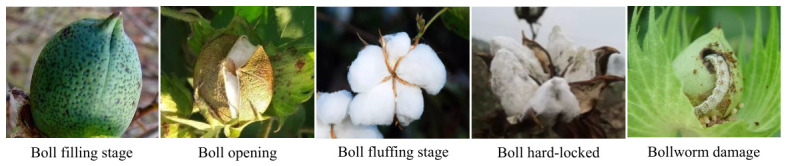
Representative examples of cotton boll states used in the dataset, including boll filling stage, boll opening, boll fluffing stage, boll hard-locked, and bollworm damage.

Data were collected from cotton experimental fields and production demonstration fields in Wuyuan County, Bayannur City, Inner Mongolia Autonomous Region, China. The collection period extended from July to October 2025 and covered the main late growth stages of cotton, including boll filling, boll opening, boll fluffing, and the preharvest stage. The dataset composition is summarized in Table 1, and representative examples of the five cotton boll states are shown in Figure 1. Visual images were acquired using close-range field imaging. Handheld cameras, fixed field imaging devices, and mobile-platform imaging systems were used to cover different shooting distances, viewpoints, illumination conditions, and occlusion levels. Handheld images were collected with a Canon EOS 90D digital single-lens reflex camera (Canon Inc., Tokyo, Japan) equipped with an EF-S 18–135 mm f/3.5–5.6 IS USM lens (Canon Inc., Tokyo, Japan). The image resolution was 6960×4640 pixels. Fixed field images were captured using a Hikvision DS-2CD3T87EWDV3-L network camera (Hangzhou Hikvision Digital Technology Co., Ltd., Hangzhou, China), which recorded boll appearance changes at the same monitoring point across different dates and illumination conditions. Mobile-platform images were obtained using a field mobile bracket equipped with a DJI Osmo Action 4 camera (DJI, Shenzhen, China). These images supplemented samples from different plant-row positions, shooting angles, and partial occlusion conditions. Five representative cotton boll states were included in image acquisition: boll filling stage, boll opening, boll fluffing stage, boll hard-locked, and bollworm damage. Boll filling samples were mainly taken from unopened green or light-green cotton bolls. Surface texture, boll fullness, and ridge structure were used to characterize the state in which lint development had not yet been exposed. Boll opening samples were collected from bolls with cracked shells and partially exposed lint, indicating the transition toward maturity. Boll fluffing samples were collected from bolls with fully exposed lint, high whiteness, and clear segmental structures. Boll hard-locked samples were obtained from bolls with incomplete opening, stiff lint, or abnormal fluffing caused by stress conditions. Bollworm damage samples were collected from bolls showing insect holes, feeding traces, insect residues, local browning, or tissue necrosis. These abnormal samples were included to help the model distinguish normal maturity progression from quality-risk symptoms.

Image acquisition and environmental monitoring were conducted from July to October 2025, but they followed different recording intervals. Handheld and mobile-platform images were collected every 3–5 days during the boll filling and boll opening stages and every 1–3 days during the boll fluffing and preharvest stages. Additional images were collected within 24 h after rainfall, irrigation, pesticide application, or visible pest and disease occurrence. Fixed image monitoring nodes captured images between 08:00 and 18:00. Each node consisted of a Hikvision DS-2CD3T87EWDV3-L RGB network camera, weatherproof housing, a mounting pole, a solar-powered battery unit, a local storage card, and a wireless communication module. The camera was installed at approximately 1.2–1.5 m above the ground and oriented toward representative cotton plants in the monitoring plot. Each image was stored with the device ID, plot ID, acquisition time, and camera position. Raw images were buffered locally and exported periodically for annotation and model training, while compressed previews and metadata were transmitted to the field gateway for inspection. Images with severe motion blur, strong backlight, raindrops on the lens, or incomplete boll visibility were removed during quality control.

**Figure 2:**
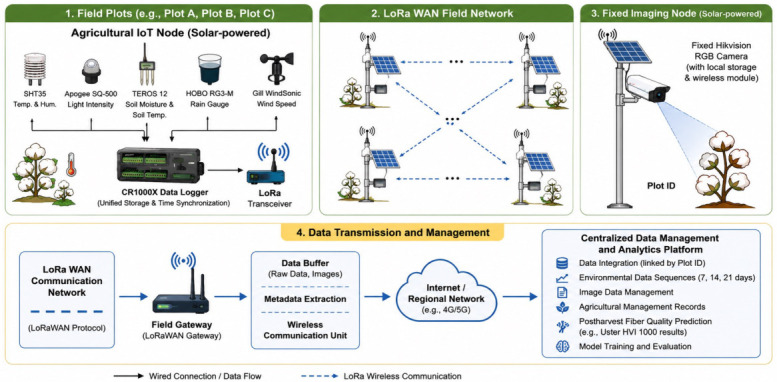
IoT environmental sensing architecture used for field data acquisition. Plot-level agricultural IoT nodes collect environmental variables through multiple sensors, store records in a local buffer, and transmit packets through a LoRa/LoRaWAN star network. The field gateway and CR1000X data logger support data aggregation, time synchronization, and database integration for subsequent multimodal modeling.

The environmental sensing architecture used a plot-level IoT topology, as illustrated in Figure 2. Each agricultural IoT node was installed in a plot-level sampling unit and connected to multiple sensors. Air temperature and relative humidity were measured using Sensirion SHT35 sensors, light intensity using an Apogee SQ-500 quantum sensor, soil moisture and soil temperature using a TEROS 12 sensor, rainfall using a HOBO RG3-M tipping-bucket rain gauge, and wind speed using a Gill WindSonic two-dimensional ultrasonic wind sensor. The node included sensor interfaces, a microcontroller, a LoRa/LoRaWAN transceiver, a local data buffer, and a power module. Environmental variables were sampled hourly. When field communication was unstable, raw records were temporarily stored in the local buffer and transmitted after the wireless link was restored.

The field nodes formed a star-type LoRa/LoRaWAN network. Sensor packets from different plots were transmitted to the field gateway and then forwarded to the CR1000X data logger for unified storage and time synchronization. The gateway performed packet reception, metadata extraction, and preliminary integrity checking. The server-side database then integrated environmental sequences, image records, agricultural management records, and postharvest fiber quality measurements according to plot ID and sampling date. For each image sampling date, environmental sequences from the preceding 7, 14, and 21 days were extracted and matched with the corresponding image records.

To reduce the influence of spatial heterogeneity in medium- and large-sized cotton fields, the field was divided into plot-level sampling units according to planting layout, irrigation regime, soil condition, and observed pest or disease status. Each sampling unit was assigned a plot ID, which was used to link image records, sensor sequences, management records, and postharvest quality data. Images from different rows, plant positions, camera viewpoints, and local backgrounds were included to cover within-field variation. Pest- or disease-related symptoms were recorded as sample metadata, and plot-level grouping was used during model evaluation to avoid assigning samples from the same plot or harvest batch to both training and test sets. Agricultural management records included irrigation, fertilization, pest and disease control, and harvesting operations. Postharvest fiber quality was measured after harvest from September to October 2025 using an USTER HVI 1000 high-volume fiber testing system for fiber length, strength, micronaire, uniformity, and maturity. Moisture regain and trash content were measured by laboratory testing and manual verification. Since fiber quality cannot be reliably assigned to individual bolls, images, sensor data, management records, and quality indicators were matched at the plot or harvest-batch level.

### 3.2. Data Augmentation

**Figure 3:**
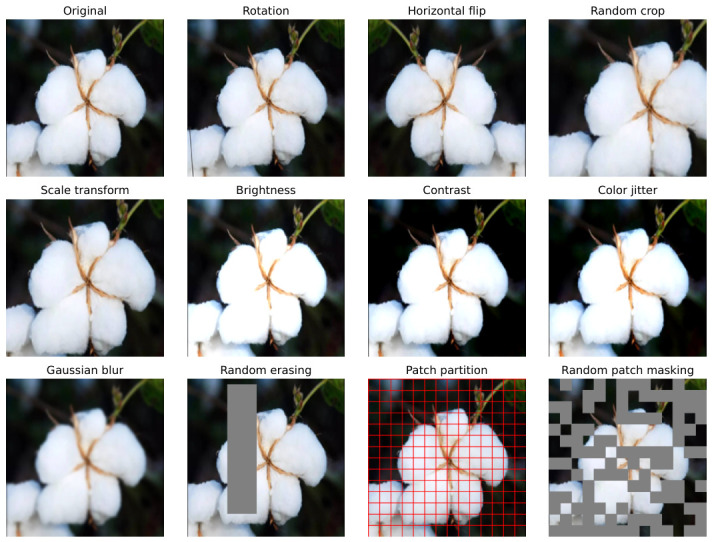
Examples of cotton boll image augmentation, patch partitioning, and random patch masking. The first ten panels show common visual augmentations used for field images. The patch partition panel shows the division of a resized image into local patches. The random patch masking panel shows the masked regions used for self-supervised visual representation learning.

To improve robustness under field conditions, augmentation was applied to cotton boll images, environmental sensor sequences, and multimodal sample pairs. Cotton boll images are affected by illumination changes, leaf occlusion, shooting angle, background weeds, and blurred lint boundaries. Therefore, visual augmentation included geometric perturbation, illumination adjustment, color perturbation, blur, and local occlusion simulation. For an original cotton boll image I, the augmented image was generated by a random operator $T_{v}\left(\cdot \right)$:

(1) $$I^{aug}=T_{v}\left(I;\theta_{v}\right),$$

where $\theta_{v}$ denotes the sampled augmentation parameters. The operator included rotation, horizontal flipping, random cropping, scale transformation, brightness adjustment, contrast adjustment, color jittering, Gaussian blurring, and random erasing. Examples of these operations are shown in Figure 3. Geometric operations simulate changes in shooting distance and viewing angle. Illumination and color operations simulate sunny, cloudy, shaded, and backlit conditions. Random erasing simulates partial occlusion by leaves, branches, or missing boll regions. The color perturbation range was limited because lint whiteness, boll-opening edges, and boll shell color are maturity-related visual cues.

For masked image modeling, each image was first resized to 224×224 pixels and divided into non-overlapping local patches. When the patch size was set to 16×16 pixels, each image was divided into 14×14 patches. A random subset of patches was then masked before being sent to the visual encoder. The decoder was trained to reconstruct the missing image regions from the visible patches. This design forced the model to infer local boll structure from surrounding context. For example, when patches covering part of a cracked shell or exposed lint were masked, the model learned to use adjacent shell edges, lint texture, and boll segment structure to reconstruct the missing regions. This process helped the visual encoder learn implicit representations related to boll cracking, fluffing degree, lint exposure, and maturity-related surface texture.

For agricultural sensor time series, augmentation was used to simulate sensor noise, short-term missing values, variable failure, and temporal-scale perturbation. Let the environmental sensor sequence be denoted by $X\in R^{T\times d}$, where *T* represents the temporal window length and *d* represents the number of environmental variables. The augmented sequence can be written as

(2) $$X^{aug}=T_{s}\left(X;\theta_{s}\right),$$

where $T_{s}\left(\cdot \right)$ includes random noise injection, time-step masking, variable masking, local temporal-window cropping, and temporal-scale perturbation. Noise injection was used to imitate sensor measurement errors:

(3) $$\tilde{X}=X+\epsilon ,\;\epsilon ∼N\left(0,\sigma^{2}\right),$$

where ϵ denotes the Gaussian perturbation term and σ denotes the noise intensity. Time-step masking and variable masking were implemented through a random mask matrix $M\in {\left\{0,1\right\}}^{T\times d}$:

(4) $$\tilde{X}=X⊙M,$$

where ⊙ denotes element-wise multiplication. This strategy simulates communication interruption, local sensor failure, and short-term data loss during field monitoring. It also encourages the model to learn temporal representations from incomplete environmental observations.

During multimodal self-supervised pretraining, visual images and environmental sequences were jointly augmented to construct positive and negative sample pairs. Cotton boll images and environmental sequences collected from the same sampling point, plot, or adjacent acquisition time window were treated as positive pairs after augmentation. Samples from different plots, growth stages, or maturity states were treated as negative pairs. Let the visual augmented view and environmental augmented sequence be denoted by $I_{i}^{aug}$ and $X_{i}^{aug}$, respectively, and their encoded representations be denoted by $z_{v,i}$ and $z_{s,i}$. The objective was to keep paired image and sensor representations close under different perturbations:

(5) $$\mathrm{sim}\left(z_{v,i},z_{s,i}\right)>\mathrm{sim}\left(z_{v,i},z_{s,j}\right),\;i\neq j.$$

These augmentation strategies allowed the model to learn from illumination variation, occlusion, background changes, sensor noise, missing observations, and temporal-window perturbation. They also strengthened the consistency between cotton boll visual traits and environmental dynamics for maturity recognition, harvest-window determination, and fiber quality prediction.

### 3.3. Proposed Method

#### 3.3.1. Overall

The proposed self-supervised multimodal deep sensing framework is built around a unified objective: modeling the pathway from cotton boll visual phenotype and environmental temporal driving factors to postharvest fiber quality prediction. The inputs include aligned cotton boll image sequences and multivariate agricultural sensor sequences within the corresponding temporal windows. Cross-modal joint representation learning is implemented in a shared encoding space. Cotton boll images are first processed by the visual phenotype self-supervised encoding module. In this module, a masked reconstruction-based pretraining strategy is used. The input image is divided into local patches, and part of the image regions is randomly masked. The encoder extracts high-dimensional representations from the unmasked regions, while the decoder reconstructs the missing visual content. Through this process, the model learns implicit representations of boll cracking structure, fluffing degree, and maturity-related texture. This process can be described as a representation learning function that minimizes reconstruction error, so that visual feature vectors are mapped into a maturity-related semantic space. At the same time, agricultural sensor time series are fed into the temporal masked modeling module. Multivariate environmental signals are represented as vector sequences that evolve over time. By randomly masking partial time steps or variable dimensions and using a temporal encoder to predict the missing values, the model learns dynamic dependencies among temperature, water availability, light, and other variables, and produces growth-stage-aware environmental embeddings. In essence, this process constrains the model to learn the temporal consistency of the conditional probability distribution. The visual and environmental representations are then introduced into the cross-modal contrastive fusion module. In the self-supervised stage, visual–environmental positive pairs are constructed from the same plot or the same temporal window. Contrastive learning is used to reduce the distance between joint representations and to increase the distance between samples from different plots or different maturity states. In this way, the correspondence between visual phenotypes and environmental driving factors is established in a unified embedding space. In the supervised fine-tuning stage, a cross-modal Transformer structure is further incorporated into the fusion module. Interactions between visual tokens and temporal tokens are realized through a cross-attention mechanism, which allows environmental variations to conditionally modulate visual maturity representations and finally generate a unified multimodal representation vector. This representation is fed into several task-specific prediction heads to simultaneously output cotton boll maturity grades, harvesting window categories, and continuous postharvest fiber quality indicators. Classification tasks are performed through softmax mapping for discrete decision-making, while regression tasks are implemented through continuous mapping heads for predicting quality distribution parameters. Overall, the framework forms an end-to-end closed-loop structure from local visual perception to temporal environmental modeling, cross-modal semantic alignment, and multitask prediction. It enables the model to learn cotton boll maturity evolution patterns and fiber quality formation mechanisms under weakly labeled or even unlabeled conditions.

#### 3.3.2. Cotton Boll Visual Phenotype Self-Supervised Encoding Module

**Figure 4:**
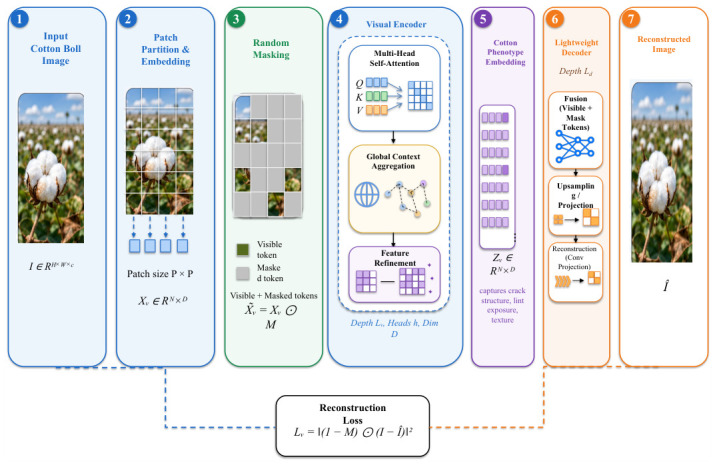
Architecture of the cotton boll visual phenotype self-supervised encoding module, which learns maturity-related structural representations through patch embedding, random masking, visual encoding, and image reconstruction.

The cotton boll visual phenotype self-supervised encoding module uses cotton boll images as input and learns visual representations constrained by structural consistency and maturity semantics through masked image modeling. As shown in Figure 4, the input image is denoted by $I\in R^{H\times W\times C}$. The image is first divided into a set of local patches through a patch partition operation and is then mapped into a token sequence $X_{v}\in R^{N\times D}$, where *N* denotes the number of patches and *D* denotes the embedding dimension. This process is written as

(6) $$X_{v}=\mathrm{PatchEmbed}\left(I\right)=\mathrm{Flatten}\left(P\left(I\right)\right)W_{v}+b_{v},$$

where P(·) denotes the patch partition operator, and $W_{v}$ denotes the linear projection matrix.

In the structural design, the visual encoder is constructed with a Transformer architecture containing $L_{v}$ stacked layers. Each layer includes a multi-head self-attention module and a feed-forward network module. Let the input of the *l*-th layer be $X_{v}^{\left(l-1\right)}$. The update process is given by

(7) $$Z_{v}^{\left(l\right)}=X_{v}^{\left(l-1\right)}+\mathrm{MSA}\left(\mathrm{LN}(X_{v}^{\left(l-1\right)})\right),$$

(8) $$X_{v}^{\left(l\right)}=Z_{v}^{\left(l\right)}+\mathrm{FFN}\left(\mathrm{LN}(Z_{v}^{\left(l\right)})\right),$$

where MSA(·) denotes the multi-head attention mechanism, whose core computation is

(9) $$\mathrm{Attention}\left(Q,K,V\right)=\mathrm{softmax}\left(\frac{QK^{⊤}}{\sqrt{d_{h}}}\right)V.$$

This structure allows the model to capture global structural dependencies among different cotton boll regions, such as the spatial relationships among boll shell crack expansion, lint exposure degree, and occluding leaves. In the decoding stage, a lightweight reconstruction network D(·) is introduced to recover the masked patches. Let the mask matrix be denoted by $M\in {0,1}^{N}$. The input is represented as ${\tilde{X}}_{v}=X_{v}⊙M$, and the decoded output is

(10) $$\hat{I}=D\left({\tilde{X}}_{v}\right).$$

The optimization objective is defined as the reconstruction error over only the masked regions:

(11) $$L_{v}={\left|\left(1-M\right)⊙\left(I-\hat{I}\right)\right|}_{2}^{2}.$$

From the perspective of information theory, this process is equivalent to maximizing the conditional likelihood of the image under partial observation, namely learning the optimal estimator of $p(I_{m}|I_{\bar{m}})$, where *m* denotes the missing region. When the reconstruction error is interpreted as the negative log-likelihood under Gaussian observation noise, this optimization can be regarded as a special case of variational lower-bound maximization, yielding

(12) $$\arg\min L_{v}\equiv \arg\max\log p_{\theta }\left(I_{m}|I_{\bar{m}}\right).$$

For structural parameter design, the input resolution (H,W) determines the number of patches *N*, the patch size controls the scale of the local receptive field, and the embedding dimension *D* determines the visual semantic capacity. The encoder depth $L_{v}$ improves cross-region structural modeling, and the number of heads *h* characterizes attention subspaces at different spatial scales. The FFN layer is usually configured as an expansion structure of D→rD→D to improve nonlinear representation ability, where *r* denotes the expansion ratio.

#### 3.3.3. Agricultural Sensor Temporal Masked Modeling Module

**Figure 5:**
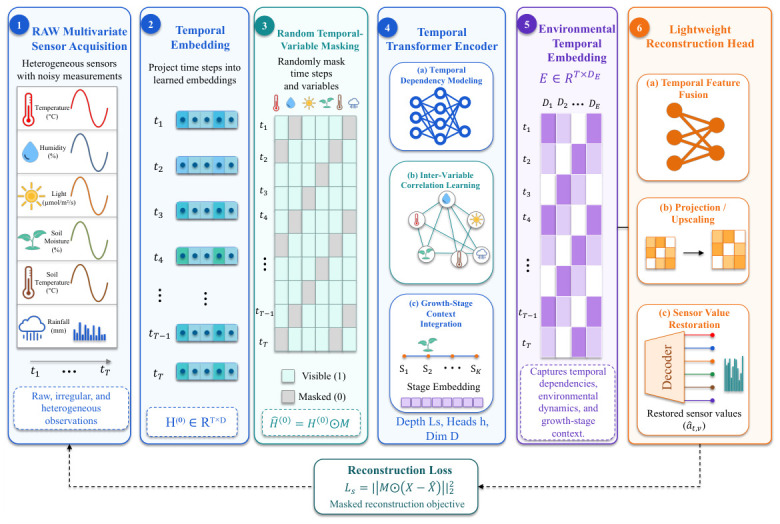
Architecture of the agricultural sensor temporal masked modeling module, which captures environmental temporal dependencies by masking and reconstructing multivariate sensor observations.

The agricultural sensor temporal masked modeling module takes multisource environmental observation sequences as input. Its core objective is to learn the dynamic environmental generation mechanism of cotton boll development from incomplete time series and to provide representations with temporal causal structure for subsequent cross-modal fusion. As shown in Figure 5, the original sensor sequence is denoted by $X\in R^{T\times d}$, where *T* denotes the time-step length and *d* denotes the dimension of multivariate environmental features. The sequence is first mapped into a hidden representation $H^{\left(0\right)}\in R^{T\times D}$ through linear embedding or one-dimensional convolution, which is formulated as

(13) $$H^{\left(0\right)}=\phi \left(XW_{e}+b_{e}\right),$$

where $W_{e}\in R^{d\times D}$ denotes the projection matrix, and ϕ(·) denotes the nonlinear activation function. A temporal mask matrix $M\in {0,1}^{T\times d}$ or ${0,1}^{T\times D}$ is then constructed, and a partially observed sequence ${\tilde{H}}^{\left(0\right)}=H^{\left(0\right)}⊙M$ is generated through Bernoulli sampling to simulate sensor missingness and incomplete observations.

In the encoding stage, an *L*-layer temporal Transformer or gated attention network is used for sequence modeling. For the *l*-th layer, the update process is expressed as

(14) $$Z^{\left(l\right)}=\mathrm{MSA}\left(H^{\left(l-1\right)}\right)+H^{\left(l-1\right)},$$

(15) $$H^{\left(l\right)}=\mathrm{FFN}\left(\mathrm{LN}(Z^{\left(l\right)})\right)+Z^{\left(l\right)},$$

where MSA(·) denotes the multi-head self-attention mechanism, and the final output is obtained through multi-head concatenation. This structure establishes long-range dependencies along the temporal dimension and captures the cumulative environmental effects during cotton boll development. The decoding task of this module is to reconstruct the masked observations. The output is defined as $\hat{X}$, which is restored from the hidden space to the original space through a linear mapping ψ(·):

(16) $$\hat{X}=\psi \left(H^{\left(L\right)}\right).$$

The optimization objective is defined as the weighted reconstruction error over only the masked regions:

(17) $$L_{rec}={\left|M⊙(X-\hat{X})\right|}_{2}^{2}.$$

From the perspective of probabilistic modeling, this task is equivalent to learning the conditional distribution $p(X_{m}|X_{\bar{m}})$, where *m* denotes the masked component. If the environmental sequence is regarded as an observational approximation of a Markov process, the reconstruction objective can be interpreted as maximizing the following likelihood lower bound:

(18) $$\log p\left(X\right)\geq E_{q(M)}\left[\log p_{\theta }\left(X_{m}|X_{\bar{m}}\right)\right],$$

where q(M) denotes the mask distribution. This formulation shows that the model approximates the marginal distribution of the real environmental dynamic system through a random missingness mechanism, thereby reducing dependence on complete observations. For structural parameter design, the input dimension *d* corresponds to the number of sensor variables, the embedding dimension *D* controls the semantic representation capacity of the environment, and the temporal window length *T* corresponds to the observational span of the cotton boll growth cycle. The encoder depth *L* improves long-term dependency modeling, and the number of heads *h* describes subspace interactions among different environmental variables. The FFN layer is generally configured as D→4D→D to enhance nonlinear expression ability, while LayerNorm is used to stabilize gradient propagation across time.

#### 3.3.4. Vision–Environment Cross-Modal Contrastive Fusion Module

The vision–environment cross-modal contrastive fusion module is used to build a unified semantic space between cotton boll visual phenotypes and environmental temporal driving factors. Its inputs are the visual encoder output $Z_{v}\in R^{N\times D}$ and the environmental encoder output $Z_{e}\in R^{T\times D}$, where *N* denotes the number of visual tokens, *T* denotes the number of time steps, and *D* denotes the shared embedding dimension. Modality projection heads are first used to map the two types of features into the same contrastive space, generating normalized representations $U_{v}=f_{v}\left(Z_{v}\right)$ and $U_{e}=f_{e}\left(Z_{e}\right)$. Each projection head is constructed with a two-layer fully connected structure, where the dimension is progressively compressed from $D\to D^{\prime }\to D^{\prime \prime }$, and nonlinear activation and normalization operations are introduced to improve discriminability.

**Figure 6:**
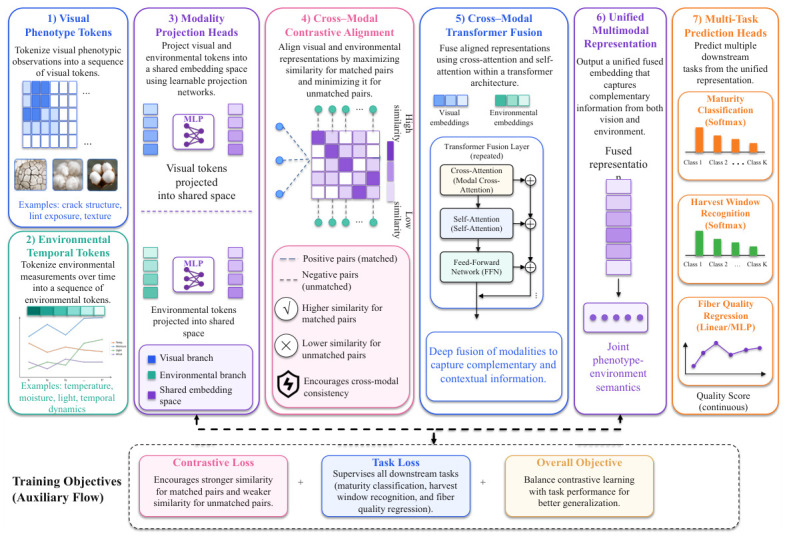
Architecture of the vision–environment cross-modal contrastive fusion module, which aligns visual phenotype tokens and environmental temporal tokens for unified multimodal prediction.

As shown in Figure 6, visual–environmental positive and negative sample pairs are constructed in the cross-modal alignment stage. For samples from the same plot or the same temporal window, their visual and environmental representations form positive pairs $\left(U_{v}^{+},U_{e}^{+}\right)$, while samples from different plots or different maturity states form negative pairs $\left(U_{v}^{-},U_{e}^{-}\right)$. The contrastive learning objective is defined as cross-modal mutual information maximization and is formulated as

(19) $$L_{c}=-\log\frac{\exp(\mathrm{sim}\left(U_{v},U_{e}\right)/\tau )}{\sum_{k}\exp\left(\mathrm{sim}\left(U_{v},U_{e}^{k}\right)/\tau \right)},$$

where sim(·) denotes the cosine similarity function, and τ denotes the temperature coefficient. This objective is equivalent to maximizing the mutual information lower bound between visual and environmental representations, namely

(20) $$I\left(U_{v};U_{e}\right)\geq E\left[\log p\left(U_{v}|U_{e}\right)\right]-\log p\left(U_{v}\right),$$

which allows the model to learn a stable cross-modal consistency structure. In the fusion structure, a cross-modal Transformer is introduced for fine-grained interaction. Let the visual tokens and environmental tokens be concatenated as a joint sequence $S=[U_{v};U_{e}]$, which is then fed into an $L_{f}$-layer cross-attention encoder. Each layer contains a dual-branch attention mechanism, where the visual queries and environmental keys and values interactively update each other. The computation is expressed as

(21) $$H_{v}^{\left(l\right)}=\mathrm{softmax}\left(\frac{Q_{v}K_{e}^{⊤}}{\sqrt{D_{h}}}\right)V_{e}.$$

This mechanism enables environmental dynamics to conditionally modulate visual maturity representations and thus realizes environment-driven visual interpretation modeling. For structural parameters, the input dimension of this module is unified as *D* by the previous two modules, and the output dimension $D^{\prime \prime }$ of the projection heads determines the capacity of the contrastive space. The number of Transformer layers $L_{f}$ controls the depth of cross-modal interaction, and the number of heads *h* describes alignment relationships in different semantic subspaces. The number of visual tokens *N* and the number of environmental time steps *T* are aligned to a shared length through unified projection or compressed into fixed vectors through pooling during the fusion stage, thereby reducing computational complexity.

## 4. Results and Discussion

### 4.1. Experimental Settings

To evaluate the proposed method in preharvest cotton boll maturity assessment and postharvest fiber quality prediction, the experiments were organized into self-supervised pretraining and supervised fine-tuning. In the pretraining stage, unlabeled cotton boll images and agricultural sensor sequences were used to initialize the visual, sensor, and cross-modal branches. The visual branch learned boll cracking structures, lint textures, and maturity-related appearance features through masked image reconstruction. The sensor branch learned temporal dependencies among temperature, humidity, light, soil moisture, and other environmental variables through masked sensor prediction. The cross-modal branch used vision–environment contrastive learning to align cotton boll phenotypes with environmental driving factors. In the fine-tuning stage, samples with maturity grades, harvest-window labels, and postharvest fiber quality labels were used for downstream prediction. To examine model performance under limited annotation, the labeled-sample proportions were set to 5%, 10%, 20%, 50%, and 100%.

For the main evaluation, a cross-plot and cross-harvest-batch split was used to reduce the influence of plot-specific background information. Samples from independent plots or harvest batches were assigned to different subsets, so that images from the same plot or harvest batch did not appear simultaneously in the training and test sets. A chronological harvest-batch split was also used as supplementary validation for temporal extrapolation. All harvest batches were first ordered by sampling and harvesting dates and denoted by $B_{1},B_{2},\ldots ,B_{N}$. The first 60% of batches were used for training, the next 20% were used for validation, and the final 20% were used for testing. Under this setting, the test set contained only later harvest batches that were not available during model training or hyperparameter selection. This design evaluates whether a model calibrated with earlier field observations can be applied to subsequent harvest batches within the same production season. Input images were resized to 224×224 pixels. Environmental sensor sequences were constructed into 7-day, 14-day, and 21-day windows according to the image acquisition dates. The model was trained with the AdamW optimizer, an initial learning rate of $1\times 10^{-4}$, a batch size of 32, and a maximum of 200 epochs. The self-supervised pretraining loss consisted of the image reconstruction loss $L_{v}$, the sensor masked reconstruction loss $L_{s}$, and the cross-modal contrastive loss $L_{c}$. During supervised fine-tuning, the objective function jointly included the maturity classification loss, the harvest-window classification loss, and the fiber quality regression loss:

(22) $$L=\lambda_{v}L_{v}+\lambda_{s}L_{s}+\lambda_{c}L_{c}+\lambda_{m}L_{m}+\lambda_{h}L_{h}+\lambda_{q}L_{q},$$

where $\lambda_{v}$, $\lambda_{s}$, $\lambda_{c}$, $\lambda_{m}$, $\lambda_{h}$, and $\lambda_{q}$ denote the weighting coefficients of the corresponding loss terms.

### 4.2. Evaluation Metrics and Baselines

For maturity grade classification, Accuracy, Precision, Recall, F1-score, Macro-F1, and AUC were used as evaluation metrics. Since the numbers of samples differed among maturity states in field acquisition, Macro-F1 was used to evaluate class-balanced recognition. For harvest-window recognition, Accuracy, Macro-F1, Recall, and the confusion matrix were reported. The recall of the suitable-harvest category was examined because missed recognition of this class may directly affect harvest scheduling. For fiber quality regression, MAE, RMSE, $R^{2}$, and MAPE were used to evaluate continuous quality indicators, including fiber length, fiber strength, micronaire value, trash content, and moisture regain. These metrics are defined as follows:

(23) $$\mathrm{MAE}=\frac{1}{n}\sum_{i=1}^{n}\left|y_{i}-{\hat{y}}_{i}\right|,$$

(24) $$\mathrm{RMSE}=\sqrt{\frac{1}{n}\sum_{i=1}^{n}{\left(y_{i}-{\hat{y}}_{i}\right)}^{2}},$$

(25) $$R^{2}=1-\frac{\sum_{i=1}^{n}{\left(y_{i}-{\hat{y}}_{i}\right)}^{2}}{\sum_{i=1}^{n}{\left(y_{i}-\bar{y}\right)}^{2}},$$

where $y_{i}$ denotes the ground-truth value, ${\hat{y}}_{i}$ denotes the predicted value, and $\bar{y}$ denotes the mean of the ground-truth values. Few-shot experiments were analyzed using different labeled-sample proportions, including 5%, 10%, 20%, 50%, and 100%. Deployment-related indicators, including the number of trainable parameters, FLOPs, single-sample inference time, and average prediction latency, were also recorded under the same test environment.

Eight representative baseline methods were selected for comparison. Random Forest [46] and XGBoost [47] represented traditional machine learning methods. Their inputs included cotton boll color, texture, cracking ratio, and statistical features derived from environmental sensor data. ResNet50 [48] and Swin Transformer [49] were used as image-only deep learning baselines. GRU [50] and Temporal Transformer [51] were used as sensor-only temporal baselines. Early Fusion [52] and Cross-Attention Fusion [53] were used as multimodal baselines. Early Fusion concatenated image features and sensor features before prediction, whereas Cross-Attention Fusion used attention-based interaction between image tokens and environmental sequence features.

All baseline models used the same training, validation, and test partitions as the proposed method. Image-based models used the same input resolution of 224×224 pixels. Sensor-based models used the same 7-day, 14-day, and 21-day environmental windows. For deep learning models, the batch size was fixed at 32, the maximum number of epochs was 200, and early stopping was applied if the validation loss did not decrease for 30 consecutive epochs. AdamW was used for neural-network baselines unless otherwise stated. The best checkpoint was selected according to the validation metric corresponding to each task. Traditional machine learning baselines were tuned on the validation set using grid search. The main training configurations are summarized in Table 2.

### 4.3. Comparison with Baseline Methods

This experiment was conducted to verify the overall advantages of the proposed method in three tasks, including cotton boll maturity classification, harvest window recognition, and postharvest fiber quality prediction, by comparing it with four types of methods: traditional machine learning, visual single-modal learning, sensor single-modal learning, and multimodal fusion.

**Figure 7:**
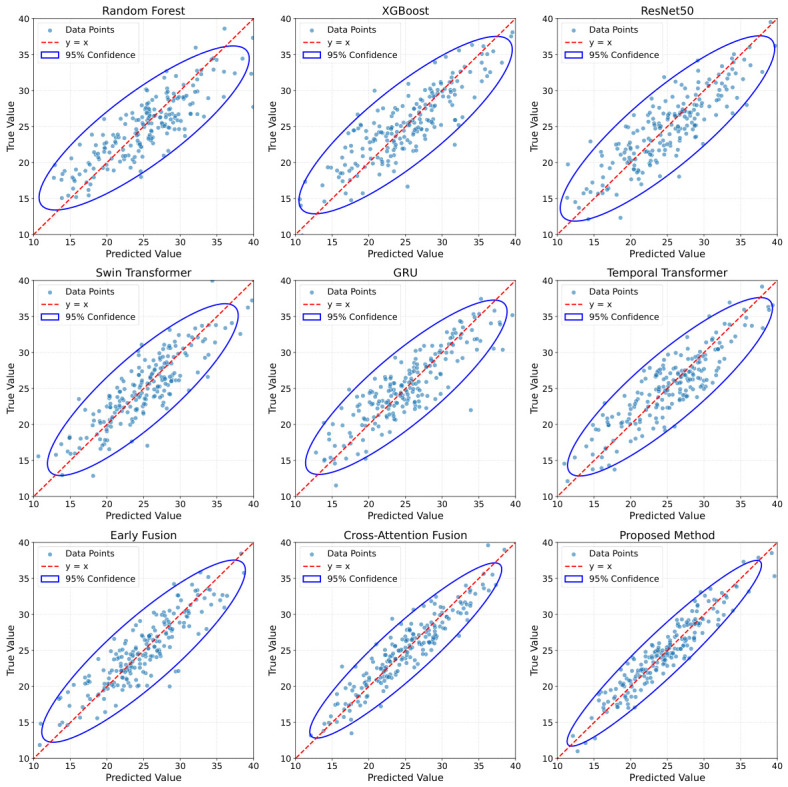
Regression fitting comparison between predicted and true fiber quality values for different baseline methods and the proposed method, where the proposed method shows the closest agreement with the ideal prediction line.

As shown in Table 3 and Figure 7, Random Forest and XGBoost could perform basic discrimination using handcrafted color features, texture features, cracking ratios, and sensor statistical features, but their overall performance was still limited. The maturity accuracy and Macro-F1 of Random Forest were 0.781 and 0.754, respectively, and the $R^{2}$ for fiber quality prediction reached only 0.681. XGBoost improved nonlinear fitting through the gradient boosting mechanism, increasing the corresponding metrics to 0.806, 0.779, and 0.714, respectively. However, its performance was still restricted by the representation capacity of handcrafted features. ResNet50 and Swin Transformer used only image information and achieved maturity accuracies of 0.842 and 0.867, respectively, showing that visual phenotypes provide important information for cotton boll maturity recognition. Swin Transformer performed better than ResNet50, mainly because its hierarchical window attention structure could better capture boll-opening edges, lint exposure, and local texture differences. GRU and Temporal Transformer used only sensor sequences, and their maturity recognition performance was lower than that of visual models. However, they achieved relatively stable results in fiber quality prediction. Temporal Transformer reached a Fiber $R^{2}$ of 0.779, suggesting that cumulative environmental effects provide important explanatory information for fiber quality formation.

The performance gaps among different models can be explained by differences in representation space, dependency modeling strategy, and modality interaction capability. Traditional machine learning methods rely on fixed handcrafted features. In essence, they partition or combine a limited set of statistical descriptors, making it difficult to represent complex spatial structural changes in cotton boll images or long-term dynamic relationships among environmental variables. ResNet50 has local receptive fields and hierarchical feature extraction ability, so it is suitable for identifying color, texture, and morphological features. However, it is less effective in modeling long-range regional relationships and global maturity structures. Swin Transformer extends the range of spatial dependency modeling through attention, which leads to a higher maturity Macro-F1. GRU models temporal dependencies through gated state recurrence, but long-sequence information can be compressed into a limited hidden state. By contrast, Temporal Transformer directly establishes associations among different time steps through attention weights, making it more suitable for describing the cumulative effects of temperature, water availability, and light on cotton boll development. Early Fusion directly concatenates image and sensor features and improves the metrics to 0.879 and 0.814, indicating the complementarity of multimodal information. However, simple concatenation does not provide explicit semantic alignment. Cross-Attention Fusion further models the conditional relationship between visual phenotypes and environmental sequences through attention, improving the corresponding metrics to 0.901, 0.884, and 0.842. In comparison, the proposed method achieved the best performance across all metrics, with maturity accuracy, Macro-F1, harvest window accuracy, Fiber RMSE, and Fiber $R^{2}$ reaching 0.934, 0.921, 0.897, 0.758, and 0.891, respectively. The improvement mainly comes from the enhanced cotton boll structural representations learned through visual masked modeling, the environmental temporal dependencies captured through sensor masked modeling, and the reduced semantic distance between visual and environmental representations from the same growth process through cross-modal contrastive learning. Together, these components produce a more stable and physiologically consistent joint representation.

### 4.4. Few-Shot Learning Comparison

This experiment was designed to verify the learning ability of the proposed method under label-scarce conditions. The focus was on whether self-supervised pretraining could reduce the impact of high annotation costs in cotton field data, the difficulty of manual maturity-grade interpretation, and the limited availability of large-scale postharvest fiber quality labels. Labeled sample ratios of 5%, 10%, 20%, 50%, and 100% were used, and the proposed method was compared with a purely supervised baseline.

**Figure 8:**
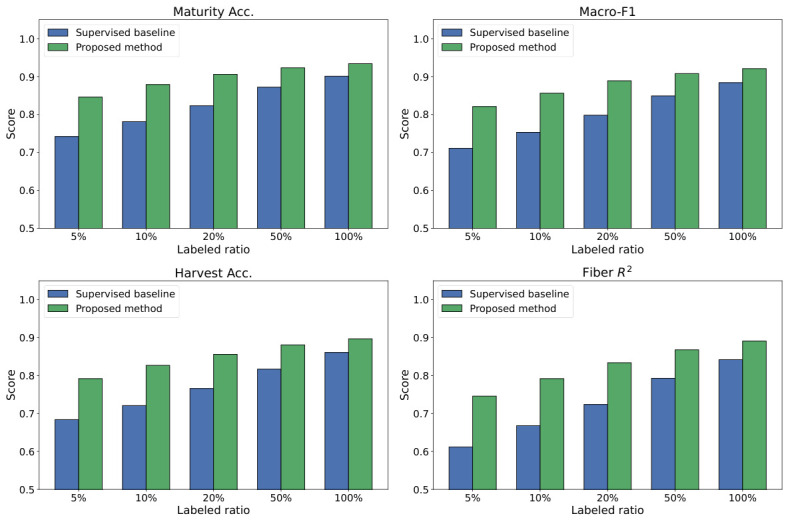
Few-shot learning comparison under different labeled-sample proportions, demonstrating that the proposed method consistently outperforms the supervised baseline under label-scarce conditions.

As shown in Table 4 and Figure 8, the proposed method clearly outperformed the supervised baseline under all labeled-sample proportions. Under the 5% labeling condition, the maturity accuracy, Macro-F1, harvest window accuracy, and Fiber $R^{2}$ of the supervised baseline were 0.742, 0.711, 0.684, and 0.612, respectively. In contrast, the proposed method reached 0.846, 0.821, 0.792, and 0.746, respectively. These results show that strong classification and regression performance can still be maintained when only a very small number of labeled samples is available. When the labeled-sample proportion increased to 10% and 20%, the proposed method continued to show clear advantages, with Fiber $R^{2}$ values of 0.792 and 0.834, respectively. This indicates that the visual structural representations and environmental temporal representations learned through self-supervised learning can be effectively transferred to fiber quality prediction. Under the 50% and 100% labeling conditions, the supervised baseline gradually approached a stable performance level, while the proposed method still achieved maturity accuracies of 0.923 and 0.934, and Fiber $R^{2}$ values of 0.868 and 0.891, respectively. This suggests that the advantages of the proposed method come not only from few-shot compensation but also from more sufficient cross-modal representation learning.

Theoretically, the supervised baseline depends entirely on limited labeled samples for parameter updates. Under low labeling ratios, it is easily affected by class imbalance, field background variation, and fiber-quality label noise. As a result, the learned decision boundary often has strong sample dependence and cannot sufficiently cover the continuous variation space of cotton boll maturity states. Before fine-tuning, the proposed method has already learned boll-opening edges, lint textures, and appearance-related maturity structures through visual masked reconstruction. It has also captured temporal dependencies among temperature, light, water availability, and other variables through sensor masked modeling. Therefore, downstream training does not start from random initialization, but from task adaptation in a more stable representation space. Mathematically, the supervised baseline mainly relies on label-driven empirical risk minimization, whereas the proposed method uses unlabeled data during pretraining to constrain the feature distribution, making image phenotypes and environmental sequences from the same growth process closer in the embedding space. This reduces variance under few-shot conditions and improves generalization ability. As the labeling ratio increased, both methods improved. However, the proposed method consistently maintained higher performance, showing that self-supervised cross-modal representations not only improve data utilization efficiency but also help the model capture more stable latent relationships between cotton boll maturity evolution and fiber quality formation.

### 4.5. Ablation Study

This ablation experiment was designed to verify the independent contribution of each key module in the proposed self-supervised multimodal deep sensing framework to cotton boll maturity recognition, harvest window determination, and postharvest fiber quality prediction.

**Figure 9:**
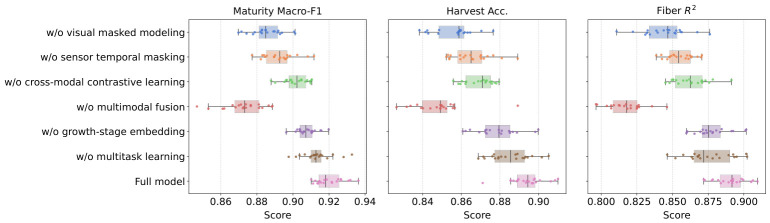
Ablation analysis of the proposed framework across maturity classification, harvest window recognition, and fiber quality prediction, showing that the full model achieves the most stable and superior performance.

As shown in Table 5 and Figure 9, when visual masked modeling was removed, Maturity Macro-F1, Harvest Acc., and Fiber $R^{2}$ decreased to 0.887, 0.856, and 0.842, respectively. This indicates that boll-opening boundaries, lint exposure textures, and maturity-related appearance structures are important cues for determining maturity status and harvest windows. When sensor temporal masked modeling was removed, the three metrics decreased to 0.895, 0.862, and 0.851, respectively, showing that environmental temporal information, especially the cumulative changes in temperature, water availability, and light, provides important explanatory information for fiber quality formation and maturity progression. When cross-modal contrastive learning was removed, the performance decreased to 0.902, 0.871, and 0.862, suggesting that retaining only visual and sensor encoders is still not enough to establish stable semantic correspondences between the two modalities. The largest performance drop appeared after multimodal fusion was removed, with Maturity Macro-F1 decreasing to 0.873 and Fiber $R^{2}$ decreasing to 0.821. This shows that independent modal representations alone are insufficient to characterize the joint relationship between cotton boll appearance changes and environmental driving factors. After removing growth-stage embedding and multitask learning, the metrics decreased to 0.908/0.879/0.871 and 0.913/0.884/0.876, respectively, indicating that stage information and shared inter-task representations further improve model stability. The full model achieved the best performance across all metrics, with Maturity Macro-F1, Harvest Acc., and Fiber $R^{2}$ reaching 0.921, 0.897, and 0.891, respectively. These results verify the effectiveness of the collaborative design of all modules.

From a theoretical perspective, the performance differences among modules mainly come from their different mechanisms for modeling data distributions, feature dependencies, and task constraints. Visual masked modeling forces the model to learn conditional relationships between local cotton boll structures and global morphology by masking partial image regions and reconstructing missing content. Therefore, it can obtain more stable maturity-related semantic representations than ordinary supervised visual features. When this module is removed, the model is more likely to rely on shallow features such as color or background, which reduces classification stability under complex field conditions. Sensor temporal masked modeling essentially learns temporal dependencies among environmental variables through missing-value prediction, allowing the model to recover the dynamic state of the growth process from incomplete observations. Without this mechanism, environmental sequence representations tend to directly fit observed values and become less capable of capturing long-term cumulative effects. Cross-modal contrastive learning pulls visual and environmental representations from the same plot or the same temporal window closer in the shared space, forming semantic consistency between the two modalities. Without this module, visual and environmental features may still coexist, but explicit alignment constraints are lacking. The clear performance decline caused by removing multimodal fusion shows that cotton boll maturation and fiber quality are not determined by a single modality, but by the joint effects of visual phenotypes and environmental processes. Growth-stage embedding provides temporal priors, allowing the same visual state to be interpreted differently under different developmental stages. Multitask learning strengthens the constraint relationships among maturity recognition, harvest determination, and quality prediction by sharing a latent space across classification and regression tasks. Therefore, the full model can jointly exploit structural reconstruction, temporal completion, cross-modal alignment, and task-coordinated optimization, resulting in a joint representation with stronger physiological consistency and generalization ability.

### 4.6. Discussion

The results indicate that boll maturity, harvest-window recognition, and fiber quality prediction are related tasks. They describe different stages of the same crop development process. In cotton production, field managers need to know which plots are ready for harvest and which plots still require waiting. They also need to identify areas with possible quality risks caused by bollworm damage, drought, rainfall, or uneven water and fertilizer management. Manual inspection can provide useful field information, but it is limited by labor availability, inspection frequency, field size, and observer experience. In large cotton-growing areas, plant-by-plant checking is inefficient. It may also miss maturity differences among plots. Incorrect harvest timing can affect mechanical harvesting efficiency, trash content, cleaning cost, fiber grade, and final economic return. Early harvesting may reduce fiber maturity and strength. Late harvesting may increase lint loss, rainfall contamination, and impurity content. The proposed method combines boll images with environmental records. Images provide direct visual evidence, such as boll-opening edges, lint exposure, color changes, and insect damage. These traits are closely related to maturity grading and harvest-window recognition. Sensor records describe the recent field environment, including temperature, humidity, light, soil moisture, and rainfall. These variables do not show boll status directly, but they affect fiber development, fluffing progress, and final fiber quality. The self-supervised training strategy uses unlabeled images and sensor sequences before task-specific fine-tuning. This is useful because detailed maturity labels and fiber quality measurements are costly to obtain. Fiber quality labels are usually available only at the plot or batch level. The fusion module links images and sensor records from the same plot and time window. This helps the model use both current visual status and previous environmental conditions, rather than relying only on background color or single-time-point appearance. The method may be used with mobile inspection devices, fixed camera systems, or farm management platforms. Its outputs can be converted into plot-level maturity maps, harvest-priority lists, and quality-risk warnings. These results may help growers arrange defoliation, ripening treatment, mechanical harvesting, and batch-wise storage. For cooperatives and large farms, the method may also support quality traceability and graded sales. Cotton batches with different quality risks can be managed separately during harvest and storage. This may reduce quality mixing and improve the consistency of batch-level cotton management. The current results therefore show the potential of combining preharvest sensing with postharvest quality prediction, while field deployment still requires further validation under more production conditions.

### 4.7. Limitations and Future Work

This study developed a self-supervised multimodal sensing framework for cotton quality assessment. The model performed well in maturity recognition, harvest-window detection, and fiber quality prediction. However, several limitations remain. First, the data were mainly collected from experimental fields and production demonstration fields in Wuyuan County, Bayannur City, Inner Mongolia. The dataset included boll filling, boll opening, boll fluffing, hard-locked bolls, and bollworm damage. Even so, model generalization still needs to be tested in other ecological regions, cotton cultivars, cultivation systems, and extreme weather conditions. Second, postharvest fiber quality testing was performed at the plot or harvest-batch level. It was therefore difficult to build a one-to-one match between an individual boll image and a fiber quality measurement. A plot-level or batch-level matching strategy was used in this study. This strategy is practical for field experiments, but it may introduce label noise. Future work should include more precise sampling localization, individual-plant tracking, and improved postharvest sample traceability. These steps would improve the match between preharvest boll traits and postharvest fiber quality. Future studies should also expand the dataset across regions, years, and cultivars. Additional data sources may be included, such as multispectral images, thermal infrared images, unmanned aerial vehicle observations, and mechanical harvesting records. These data could help extend the model from plot-level prediction to field-scale zoned harvesting and quality-graded storage. Further work is also needed to evaluate model stability on edge devices and farm management platforms under routine production conditions.

## 5. Conclusions

A self-supervised multimodal sensing framework was developed for cotton quality assessment. The framework links preharvest boll images, environmental sensor records, and postharvest fiber quality measurements. It uses three main components: an image encoder, a sensor encoder, and a visual–environmental fusion module. The image encoder learns boll structure from masked image reconstruction. The sensor encoder learns temporal patterns from masked sensor reconstruction. The fusion module aligns visual traits with environmental conditions for downstream prediction. The experiments evaluated three tasks: maturity classification, harvest-window recognition, and fiber quality prediction. The proposed method achieved higher mean performance than the tested machine learning, single-modal deep learning, and multimodal fusion baselines. Few-shot results showed that self-supervised pretraining was useful when labeled samples were limited. Ablation results showed that visual masking, sensor masking, contrastive alignment, and multimodal fusion each contributed to the final performance. These findings support the use of paired image and sensor data for preharvest cotton assessment. They also suggest that the method can provide useful information for harvest scheduling and batch-level quality management.

## Figures and Tables

**Table 1:** Dataset composition for preharvest cotton boll maturity assessment and postharvest fiber quality prediction.

Data Type	Quantity	Main Annotation/Indicator
Boll filling-stage images	4200	Closed boll morphology
Boll opening images	4600	Shell cracking degree
Boll fluffing-stage images	5100	Lint exposure status
Boll hard-locked images	2300	Abnormal fluffing status
Bollworm damage images	2000	Insect damage features
Environmental sensor data	18,720	Temperature, humidity, light, and water dynamics
Agricultural management records	320	Irrigation, fertilization, and pesticide operations
Postharvest fiber quality data	320	Length, strength, and micronaire

**Table 2:** Training configurations and model complexity of the proposed method and baseline models.

Method	Modality	Parameters	Epochs	Optimizer	Main Hyperparameters
Random Forest [46]	Handcrafted + sensor statistics	N/A	N/A	N/A	500 trees, maximum depth 20, minimum samples per leaf 2
XGBoost [47]	Handcrafted + sensor statistics	N/A	N/A	N/A	500 estimators, maximum depth 6, learning rate 0.05, subsample 0.8, column sample ratio 0.8
ResNet50 [48]	Image	25.6 M	200	AdamW	Learning rate $1\times 10^{-4}$, weight decay $1\times 10^{-4}$, batch size 32
Swin Transformer [49]	Image	28.3 M	200	AdamW	Learning rate $5\times 10^{-5}$, weight decay $1\times 10^{-4}$, batch size 32
GRU [50]	Sensor sequence	0.21 M	200	AdamW	Two GRU layers, hidden size 128, learning rate $1\times 10^{-3}$, batch size 32
Temporal Transformer [51]	Sensor sequence	1.46 M	200	AdamW	Four Transformer layers, 4 attention heads, hidden dimension 128, learning rate $1\times 10^{-4}$
Early Fusion [52]	Image + sensor	26.0 M	200	AdamW	ResNet50 image encoder, sensor MLP, feature concatenation, learning rate $1\times 10^{-4}$
Cross-Attention Fusion [53]	Image + sensor	29.1 M	200	AdamW	Cross-attention fusion with 4 heads, hidden dimension 256, learning rate $1\times 10^{-4}$
Proposed method	Image + sensor	31.8 M	200	AdamW	Mask ratio 0.5 for image patches, sensor masking ratio 0.3, contrastive temperature 0.07, learning rate $1\times 10^{-4}$

**Table 3:** Comparison with baseline methods on cotton boll maturity classification, harvest-window recognition, and postharvest fiber quality prediction. Results are reported as mean ± standard deviation over five runs.

Method	Modality	Mat. Acc.	Mat. Macro-F1	Harv. Acc.	Fiber RMSE	Fiber $R^{2}$
Random Forest	Handcrafted + Sensor	0.781±0.010	0.754±0.012	0.736±0.014	1.284±0.035	0.681±0.018
XGBoost	Handcrafted + Sensor	0.806±0.009	0.779±0.011	0.758±0.013	1.196±0.032	0.714±0.017
ResNet50	Image	0.842±0.008	0.818±0.010	0.793±0.012	1.142±0.030	0.738±0.016
Swin Transformer	Image	0.867±0.009	0.846±0.010	0.819±0.011	1.081±0.028	0.761±0.015
GRU	Sensor	0.798±0.011	0.771±0.012	0.766±0.013	1.105±0.031	0.752±0.016
Temporal Transformer	Sensor	0.824±0.010	0.801±0.011	0.789±0.012	1.047±0.029	0.779±0.015
Early Fusion	Image + Sensor	0.879±0.008	0.858±0.009	0.836±0.010	0.962±0.026	0.814±0.014
Cross-Attention Fusion	Image + Sensor	0.901±0.008	0.884±0.009	0.861±0.010	0.894±0.027	0.842±0.013
Proposed Method	Image + Sensor	0.934±0.006	0.921±0.007	0.897±0.008	0.758±0.024	0.891±0.012
*p*-value (Proposed Method vs Cross-Attention Fusion)		0.002	0.003	0.004	0.001	0.002

**Table 4:** Few-shot learning comparison under different proportions of labeled samples. Results are reported as mean ± standard deviation over five runs.

Labeled Ratio	Method	Mat. Acc.	Mat. F1	Harv. Acc.	Fiber $R^{2}$
5%	Supervised baseline	0.742±0.018	0.711±0.020	0.684±0.022	0.612±0.025
5%	Proposed method	0.846±0.012	0.821±0.014	0.792±0.016	0.746±0.021
5%	*p*-value	0.001	0.001	0.002	0.001
10%	Supervised baseline	0.781±0.016	0.753±0.018	0.721±0.020	0.668±0.023
10%	Proposed method	0.879±0.011	0.856±0.013	0.827±0.015	0.792±0.019
10%	*p*-value	0.002	0.002	0.002	0.001
20%	Supervised baseline	0.823±0.014	0.798±0.016	0.766±0.018	0.724±0.021
20%	Proposed method	0.906±0.010	0.889±0.012	0.856±0.014	0.834±0.017
20%	*p*-value	0.003	0.003	0.004	0.002
50%	Supervised baseline	0.872±0.012	0.849±0.014	0.817±0.016	0.793±0.018
50%	Proposed method	0.923±0.008	0.908±0.010	0.881±0.012	0.868±0.015
50%	*p*-value	0.006	0.005	0.004	0.003
100%	Supervised baseline	0.901±0.008	0.884±0.009	0.861±0.010	0.842±0.013
100%	Proposed method	0.934±0.006	0.921±0.007	0.897±0.008	0.891±0.012
100%	*p*-value	0.008	0.006	0.005	0.004

**Table 5:** Ablation study of the proposed self-supervised multimodal deep sensing framework. Results are reported as mean ± standard deviation over five runs.

Variant	V-SSL	S-Mask	C-Fusion	Mat. Macro-F1	Harv. Acc.	Fiber $R^{2}$
w/o visual masked modeling	×	✓	✓	0.887±0.009	0.856±0.011	0.842±0.016
w/o sensor temporal masking	✓	×	✓	0.895±0.009	0.862±0.010	0.851±0.015
w/o cross-modal contrastive learning	✓	✓	×	0.902±0.008	0.871±0.010	0.862±0.014
w/o multimodal fusion	✓	✓	×	0.873±0.010	0.843±0.012	0.821±0.017
w/o growth-stage embedding	✓	✓	✓	0.908±0.008	0.879±0.009	0.871±0.014
w/o multitask learning	✓	✓	✓	0.913±0.008	0.884±0.010	0.876±0.014
Full model				0.921±0.007	0.897±0.008	0.891±0.012
*p*-value (Full model vs. strongest ablated variant)				0.018	0.012	0.009

## Data Availability

The data presented in this study are available on request from the corresponding author.
